# Serology of Paracoccidioidomycosis Due to *Paracoccidioides lutzii*

**DOI:** 10.1371/journal.pntd.0002986

**Published:** 2014-07-17

**Authors:** Gregory Gegembauer, Leticia Mendes Araujo, Edy Firmina Pereira, Anderson Messias Rodrigues, Anamaria Mello Miranda Paniago, Rosane Christine Hahn, Zoilo Pires de Camargo

**Affiliations:** 1 Universidade Federal de São Paulo (UNIFESP), Departamento de Microbiologia, Imunologia e Parasitologia, Disciplina de Biologia Celular, São Paulo, São Paulo, Brasil; 2 Faculdade de Medicina (FAMED) da Universidade Federal de Mato Grosso do Sul (UFMS), Campo Grande, Mato Grosso do Sul, Brasil; 3 Núcleo de Doenças Infecciosas e Tropicais, Universidade Federal do Mato Grosso (UFMT), Cuiabá, Mato Grosso, Brasil; University of California San Diego School of Medicine, United States of America

## Abstract

*Paracoccidioides lutzii* is a new agent of paracoccidioidomycosis (PCM) and has its epicenter localized to the Central-West region of Brazil. Serological diagnosis of PCM caused by *P. lutzii* has not been established. This study aimed to develop new antigenic preparations from *P. lutzii* and to apply them in serological techniques to improve the diagnosis of PCM due to *P. lutzii*. *Paracoccidioides lutzii* exoantigens, cell free antigen (CFA), and a TCA-precipitated antigen were evaluated in immunodiffusion (ID) tests using a total of 89 patient sera from the Central-West region of Brazil. Seventy-two sera were defined as reactive for *P. brasiliensis* using traditional antigens (AgPbB339 and gp43). Non-reactive sera for traditional antigens (n = 17) were tested with different *P. lutzii* preparations and *P. lutzii* CFA showed 100% reactivity. ELISA was found to be a very useful test to titer anti-*P. lutzii* antibodies using *P. lutzii*-CFA preparations. Sera from patients with PCM due to *P. lutzii* presented with higher antibody titers than PCM due to *P. brasiliensis* and heterologous sera. In western blot, sera from patients with PCM due to *P. lutzii* were able to recognize antigenic molecules from the *P. lutzii*-CFA antigen, but sera from patients with PCM due to *P. brasiliensis* could not recognize any *P. lutzii* molecules. Due to the facility of preparing *P. lutzii* CFA antigens we recommend its use in immunodiffusion tests for the diagnosis of PCM due to *P. lutzii*. ELISA and western blot can be used as complementary tests.

## Author Summary

Tropical diseases, such as paracoccidioidomycosis, are the most common type of neglected diseases. From 1980 to 1995, 3,181 deaths from paracoccidioidomycosis occurred in Brazil, representing the eighth most common cause of death from predominantly chronic or recurrent types of infectious and parasitic diseases, showing considerable magnitude and low visibility. Paracoccidioidomycosis is traditionally assumed to be caused solely by *Paracoccidioides brasiliensis*, but a new species, *Paracoccidioides lutzii*, was discovered in the Central-Western region of Brazil. Thus, new antigenic preparations and tests for an accurate differential diagnosis between these two species appear to be needed. This study aimed to develop new antigenic preparations from *P. lutzii* isolates to improve the diagnosis of paracoccidioidomycosis. We used patient serum samples predominantly from the Central-Western region of Brazil. Various antigenic preparations were tested, and a cell free antigen derived from *P. lutzii* was an excellent antigen for serological diagnosis and able to diagnose 100% of sera from patients with PCM due to *P. lutzii*.

## Introduction

Paracoccidioidomycosis (PCM) is a mycotic disease caused by species of the genus *Paracoccidioides*, a group of thermally dimorphic fungi that grow in mycelial form at room temperature and as budding yeasts when cultured at 37°C or in parasitism in host tissues. PCM is limited to Latin American countries, and the most important regions of endemicity are found in Brazil, Colombia, and Venezuela [1]. PCM presents as two major clinical forms: the acute or sub-acute form and the chronic form. In Brazil, PCM is considered the eighth most common cause of death among infectious and parasitic chronic diseases, with a mortality rate of 1.45 per million population [2]. However, PCM may be considered a neglected disease because very few regions of this country have official prevention programs.

The criteria for a definitive diagnosis of PCM are based on demonstrating the presence of the fungus as multiple budding cells in clinical materials. A culture is relatively difficult to obtain, and it is a slow procedure. As an adjunct to clinical and histological findings, serological tests can help establish a diagnosis. The detection of antibodies in serum has been one of the main tools for the diagnosis of PCM and may be useful for monitoring its evolution and response to treatment. Among the different serological techniques, the double immunodiffusion (ID) test is the most commonly used and has a sensitivity of 80 to 95% [3].

Enzyme-linked immunosorbent assay (ELISA) has been employed in the serology of various mycotic diseases [4]–[8]. However, ELISA has important limitations due to cross reactivity. In an attempt to improve the specificity of ELISA for the diagnosis of PCM, Albuquerque and Camargo [9] demonstrated that different procedures are inefficient for eliminating all cross-reacting antibodies and obtaining a specific diagnosis.

Exoantigens from cultures of *P. brasiliensis* were studied by immunoblotting, and components were detected using serum from patients with PCM. Anti-*P. brasiliensis* IgG reacted with four major components of 70, 52, 43, and 20–21 kDa. The 43 kDa glycoprotein (gp43) was the predominant IgG reactive antigen and recognized by 100% of the patient sera, and the 70 kDa glycoprotein was recognized by 96% of the tested sera. Both gp43 and gp70 can be considered to be markers for human PCM [10].

**Figure 1:**
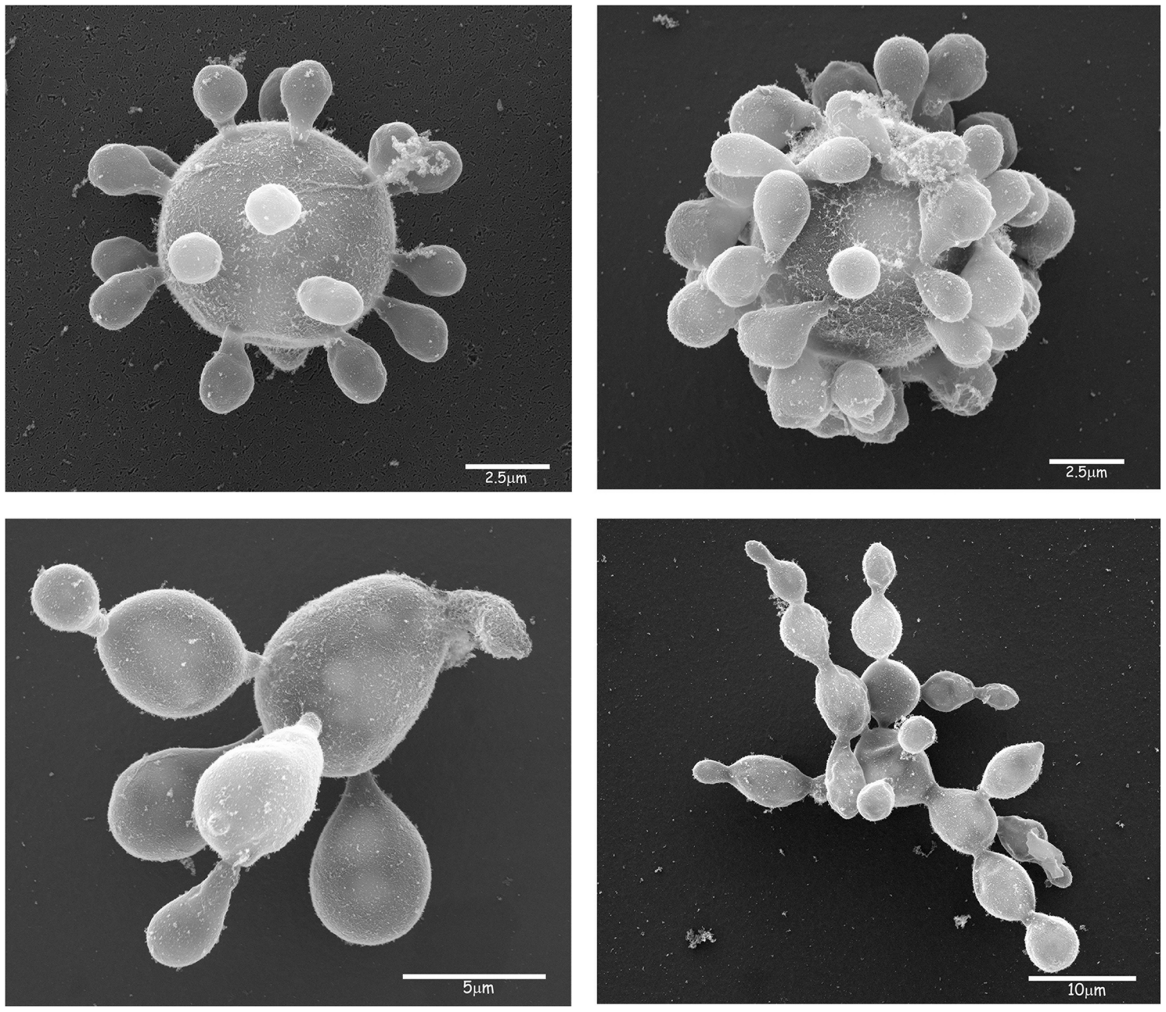
Electron microscopy of *P. lutzii* yeast phase showing multiple budding cells.

**Figure 2:**
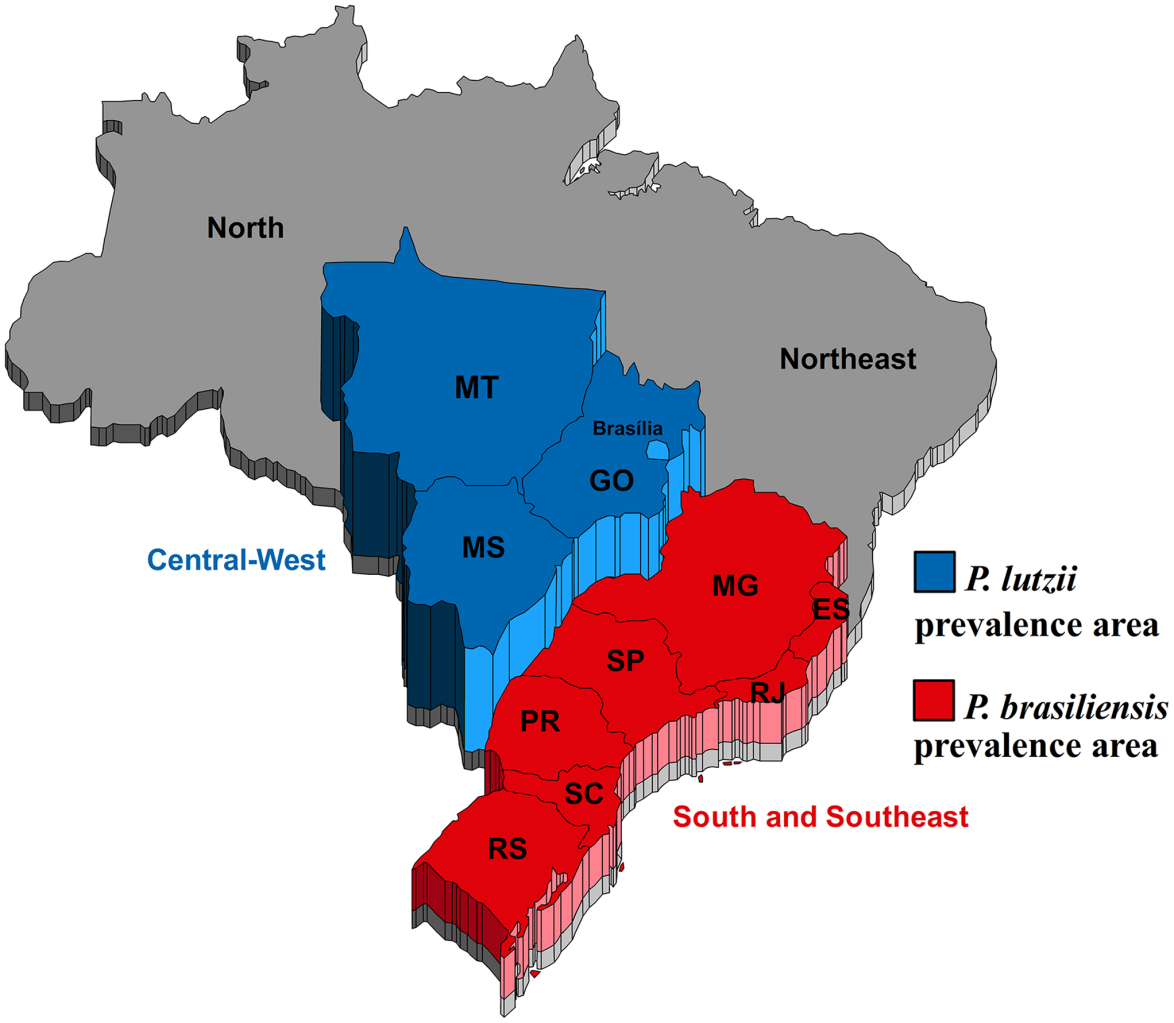
Map of the Brazilian regions, highlighting the Central - West region where *P. lutzii* predominates and the South and Southeast region where *P. brasiliensis* predominates.

Until recently, PCM was assumed to be caused solely by *P. brasiliensis* and transmitted to humans by inhalation of fungal propagules from the mycelia phase occurring in nature. Recent publications [11]–[12] support the idea of several cryptic *Paracoccidioides* species being phylogenetically related. The real incidence of each phylogenetic species and their implication on ecology and clinical practice is difficult to establish because there is a lack of information in the literature concerning the distribution of these entities. The Phylogenetic Species Recognition (PSR) method based on genealogical concordance (GCPSR) [13]–[14] has been used to detect limits in many pathogenic fungal species [15]–[17]. Using GCPSR methodology and several isolates of *P. brasiliensis* from different regions of Latin America, Matute et al. [18] concluded that *P. brasiliensis* is not a monotypic taxon, but a complex of species: phylogenetic species 1 (S1), broadly distributed through the Latin America [18]–[20], phylogenetic species 3 (PS3) is restricted to clinical cases in Colombia [18]–[20], and a few isolates in the clade phylogenetic species 2 (PS2) have been reported [18]–[20] in Brazil and Venezuela. Phylogenetic analysis of 14 genes in 21 isolates revealed that isolate Pb01 cannot be grouped into any of these species and constitutes a new clade [21]. Seventeen new isolates belonging to this fourth cryptic species (Pb01-like) have been identified, 16 of which originate in the Central-West region of Brazil, and one from Ecuador [12]. Thus, Teixeira et al. [22] proposed a new species, *Paracoccidioides lutzii*, which was formerly known as ‘Pb01-like’ strains based on phylogenetic and comparative genomics data, recombination analysis, and morphological characteristics (Fig. 1). *P. lutzii* occurs mainly in the Central-West region of Brazil, but it was also recently described in the northern regions [23]. The Central-West region of Brazil is composed of the states of Mato Grosso, Mato Grosso do Sul, and Goiás, which can be regarded as the areas with highest incidence of PCM caused by *P. lutzii*. In the South and Southeast regions, *P. brasiliensis* is the predominant species (Fig. 2).

With the introduction of dissimilar species, PCM serology has been challenging for diagnosis. Several efforts highlight the antigenic variability of this complex, which may have led to a substantial number of false negative results. To date, no studies describing the standardization of *P. lutzii* antigens for PCM serology are available. In order to improve serological parameters for the diagnosis of PCM caused by *P. lutzii*, the present study presents a strategy for the immunodiagnosis of PCM caused by *P. lutzii* by testing three types of antigenic preparations using ID, ELISA, and Western blot.

## Methods

### Ethics statement

This study was approved by the Research Ethics Committee with protocol number CAAE: 17177613.6.0000.5541 by Federal University of Mato Grosso (UFMT) and protocol number 1796-10 by Universidade Federal de São Paulo (UNIFESP). Protocol number of Universidade Federal do Mato Grosso do Sul (UFMS): 354.989. All adult subjects provided informed written consent and the study was approved by ethical committee under number 288.250/CEP/HUJM/UFMT.

### Fungal strains

Fourteen isolates of *P. lutzii* were obtained from different regions of Brazil (Table 1). Five isolates were obtained from the oral or cutaneous lesions of patients from Júlio Muller University Hospital (HUJM/UFMT) in Cuiabá, MT, in the Central-West region of Brazil. The clinical materials were collected by Dr. R. Hahn. Two isolates were obtained in the North region (Pará state) by Dr. S.H. Marques-da-Silva [23]. Three isolates were isolated in Goiás state (Central-West region) and donated by Dr. C.M.A. Soares, and four isolates were donated by Dr. E. Bagagli. *P. lutzii* was confirmed by genotyping [12].

**Table 1:** *Paracoccidioides lutzii* isolates used in the study.

EPM* code	Original Code	Species	Origen	Donated by
EPM 147	Pb01	*P. lutzii*	Goiás	Dra. C. M. A. Soares
EPM 148	8941	*P. lutzii*	Goiás	Dra. C. M. A. Soares
EPM 196	11MFC	*P. lutzii*	Mato Grosso	Dra. R. Hahn
EPM 201	1578	*P. lutzii*	Goiás	Dra. C. M. A. Soares
EPM 205	RO-SC 102810	*P. lutzii*	Rondônia	Dr. E. Bagagli
EPM 206	Pb66	*P. lutzii*	Goiás	Dr. E. Bagagli
EPM 208	EP01	*P. lutzii*	Goiás	Dr. E. Bagagli
EPM 209	Pb 8334	*P. lutzii*	Goiás	Dr. E. Bagagli
EPM 212	IEC-2005	*P. lutzii*	Pará	Dra. S. H. M. da Silva
EPM 213	IEC-1744	*P. lutzii*	Pará	Dra. S. H. M. da Silva
EPM 226	2912	*P. lutzii*	Mato Grosso	Dra. R. Hahn
EPM 223	RH9840	*P. lutzii*	Mato Grosso	Dra. R. Hahn
EPM 227	3159	*P. lutzii*	Mato Grosso	Dra. R. Hahn
EPM 228	2875	*P. lutzii*	Mato Grosso	Dra. R. Hahn

*EPM = Escola Paulista de Medicina, Universidade Federal de São Paulo, São Paulo, Brazil.

### Genotyping of *P. lutzii* isolates

Fungal DNA was extracted from suspected colonies and subjected to *HSP70* gene amplification using the primers HSPMMT1 (5′-AAC CAA CCC CCT CTG TCT TG-3′) and PLMMT1 (5′-GAA ATG GGT GGC AGT ATG GG-3′) as described by Teixeira et al. [12] in order to identify an exclusive indel region of *P. lutzii*. *P. lutzii* Pb01 was used as a positive control and *P. brasiliensis* B339 as a negative control.

### Patient sera

For the ID study, 89 serum samples from PCM patients (75 males and 14 females, age range 23–78 years) were evaluated: 55 from Mato Grosso state (Cuiabá and region, Central-West region) and 34 from Mato Grosso do Sul state (Campo Grande and region, Central-West region). All patients presented with the chronic form of the disease and exhibited clinical and laboratory signs of the disease with pulmonary system involvement and mucosal or mucocutaneous lesions. The diagnosis was endorsed by the clinical experience of the physician responsible for the patient. PCM was confirmed in most patients via direct examination of secretions, such as sputum, oral mucosa lesion samples, and biopsies. However, serological ID tests were not positive for all patients when using the traditional exoantigen from *P. brasiliensis* B339 (AgPbB339 standard antigen). As a gold standard, we used three sera from PCM patients with positive cultures for *P. lutzii* (positive control) determined by genotyping; sera and isolates were obtained by R. Hahn). Heterologous sera from patients with histoplasmosis (n = 15), sporotrichosis (n = 15), and aspergillosis (n = 15) were also tested. Finally, 15 serum samples from healthy individuals were also studied (negative control).

For ELISA, a batch of sera from Central-Western Brazil was selected. Twenty-eight serum samples from patients with PCM due to *P. lutzii* (previously confirmed by ID using *P. lutzii* antigen) and 28 serum samples from patients with PCM due to *P. brasiliensis* from São Paulo, southeast region (previously confirmed by ID using AgPbB339) were used in this assay. Among the sera from patients with PCM caused by *P. lutzii*, three lacked treatment, 16 had 6 to 12 months of therapy, and 9 had 13 to 18 months of treatment. As a gold standard or positive control, three sera from PCM patients with positive cultures for *P. lutzii* were used. Heterologous sera and negative controls were also studied.

For the Western blot assays, 12 serum samples from patients with PCM due to *P. brasiliensis* and 12 serum samples from patients with PCM due to *P. lutzii* (previously positive by ID and ELISA) were used. Heterologous sera and negative control sera were also studied.

### Antigenic preparations

#### *P. lutzii* exoantigen preparation: Culture medium and growth conditions

Laboratories working with primary fungal pathogens should handle live cultures in a biological safety cabinet. The fungus was initially grown on Sabouraud medium (SAB; Difco Laboratories) slant tubes for 3 days at 35°C. At that time, growth consisting entirely of yeast cells was collected from at least 10 tubes, yielding an inoculum of approximately 2×10^6^ cells. Cell aggregates were counted as single cells. Total counts were expressed as the mean of three independent determinations. These cells were inoculated into 500 ml of Fava Netto medium [24] in 1800-ml Fernbach flasks. The flasks were incubated in a rotator shaker (100 rpm) at 35°C for 7 days. After incubation, the culture was killed with Thimerosal (0.2 g/l final concentration) and returned to the shaker for 24 h, followed by plating on blood agar slants. The slants were incubated for at least 72 h to ensure that the cultures were non-viable. A presumptive test for viability was microscopic observation of cells suspended in 0.4% trypan blue dye. Dead cells take up the dye. Supernatant was collected following paper filtration, concentrated under vacuum at 45°C, and dialyzed against three changes of distilled water (3 liters each). The molecular weight cut-off of the dialysis membrane was 10 kDa. After dialysis, if the final volume increased, the solution was concentrated again. After this step, the antigen was tested by ID. If positive reactions were obtained with the control sera, the antigen was aliquoted and maintained at −20°C. If the reactions were negative, the antigen was concentrated again and retested. In general, a filtrate concentration of 20 to 30-fold was necessary. The control serum consisted of serum from a PCM patient infected with *P. lutzii* (gold standard). The protein concentration of the preparations was determined using the Bradford method [25]. The presence of antigenic fractions was monitored by sodium dodecyl sulfate polyacrylamide gel electrophoresis (SDS-PAGE) [26] followed by silver staining. Finally, the antigen was tested by the ID test.

#### Antigen precipitated with trichloroacetic acid (TCA) from *P. lutzii* exoantigen

The same volume of exoantigen sample (0.5 ml) was added to 20% TCA and incubated for 30 min under slow stirring at 4°C [27]. The sample was centrifuged (14,000 rpm) for 15 min at 4°C and the supernatant carefully removed. The precipitate consisted of 20 µl. Next, 200 µl of ice-cold acetone was added to the precipitate and the mixture centrifuged (14,000 rpm) for 5 min at 4°C. Finally, the supernatant was removed and the pellet allowed to dry before re-suspension in 100 µl phosphate buffered saline (PBS) to constitute the TCA-precipitate antigen.

#### Preparation of *P. lutzii* cell-free antigen (CFA)

CFA corresponds to molecules that are secreted by the fungus across the cell wall, adhere to the surface of the fungus, and then released during antigenic preparation. *P. lutzii* isolates were grown on SAB (Difco Laboratories) at 35°C for 7 days. The fungal growth was collected from five tubes by gently scraping the surface. The cell mass was suspended in 1 ml of PBS, vortexed for 60 s, and immediately centrifuged at 10,000 g in an Eppendorf tabletop centrifuge for 60 s. The supernatant contained the CFA [28]. Protease inhibitor PMSF (20 mM final concentration) was added to the CFA and finally lyophilized. Before use, each CFA preparation was resuspended in saline. The protein concentration varied between 1220 and 2000 µg/ml. All preparations were tested by ID, ELISA, and Western blot with sera from PCM patients. Control sera, such as positive sera, heterologous sera, and negative sera were also included in the study. The protein contents were determined using the Bradford method [25].

#### *P. brasiliensis* exoantigen and gp43 antigen

For the traditional *P. brasiliensis* exoantigen preparations (AgPbB339), we used *P. brasiliensis* B339 isolate as described previously [29]. Purified gp43 antigen was obtained by affinity chromatography of the crude exoantigen of *P. brasiliensis*-B339 on Sepharose 4B-IgG (Mab17c anti-gp43) (gift of Dr. R. Puccia). Gp43 was eluted from the column using 0.1 M HCl-glycine buffer (pH 2.8) and immediately neutralized with 2 M Tris (pH 9.0). The eluate was freeze-dried, re-dissolved in sodium dodecyl sulphate (SDS) sample buffer, and analyzed by SDS-PAGE.

### Immunodiffusion assays

Three millimeters of melted 1% agarose (Sigma A-6877) in PBS was poured onto a glass slide (75×25 mm). The pattern for this micro-ID test consisted of a central well surrounded by six wells, each 3 mm in diameter. The central well located 6 mm (edge-to-edge) from the other wells was filled with the antigen solution. Each slide contained two sets of wells. On each slide the two central wells were filled with 10 µl of antigen. Slides were incubated overnight in a moist chamber at room temperature (20–25°C), and then washed for 1 h in 5% sodium citrate and for 24–48 h in saline. The slides were dried, stained for 5 min with 0.15% Coomassie Brilliant blue (Sigma) in ethanol∶ acetic acid∶ water (4∶2∶4; v∶v), and destained in the solvent mixture alone, when necessary. Precipitation bands were recorded by visual observation [30].

### Using different antigenic preparations for diagnosis

Suspected PCM sera were tested first by ID using the traditional *P. brasiliensis* exoantigen (AgPbB339) and the purified gp43 antigen from *P. brasiliensis* B339 in order to discriminate sera from patients with PCM caused by *P. brasiliensis*. The non-reactive PCM sera, which became the focus of this study, were tested with the three different antigenic preparations. Scheme S1 outlines the final strategy to distinguish sera from patients with PCM caused by *P. lutzii* and *P. brasiliensis*.

### ELISA

During pilot studies, CFA preparations from different *P. lutzii* strains were tested and examined by checkerboard titration for antibody detection and *P. lutzii* strain EPM 208 was chosen for this study. The CFA was used in ELISA at 12.5 µg/ml to detect circulating anti-*P. lutzii* antibodies. We selected 28 serum samples from patients with PCM due to *P. lutzii* previously confirmed by ID using CFA from *P. lutzii* EPM 208, 28 serum samples from patients with PCM due to *P. brasiliensis* previously confirmed by ID using AgPbB339 and gp43 as antigens, heterologous and negative control sera. Serum samples from patients with PCM due to *P. lutzii* were from Cuiaba-MT and Campo Grande-MS in the Central-West region of Brazil. As a gold standard, we used three sera from PCM patients with positive cultures for *P. lutzii*. All sera from patients with PCM due to *P. brasiliensis* and heterologous sera came from Hospital São Paulo, Escola Paulista de Medicina (São Paulo, SP, Southeast region). All sera were divided into aliquots and stored at −20°C.

Polystyrene flat-bottomed plates (Costar; 96-wells) were coated with CFA (*P. lutzii* EPM 208) at 12.5 µg/ml diluted in 0.1 M carbonate buffer (pH 9.6; 100 µl/well) and incubated for 2 h at 37°C and overnight at 4°C. The remaining binding sites were blocked with PBS containing 0.1% Tween 20 (PBS-T) and 5% non-fat dry milk (PBS-T-M) (200 µl/well) for 4 h at 37°C. After washing three times with PBS-T, diluted serum (100 µl/well) (1∶50 to 1∶204,800 in PBS-T) was added for 1 h at 37°C. After washing three times with PBS-T, 100 µl of goat anti-human IgG-peroxidase (Sigma) (1∶1000 in PBS-T) was added in each well and incubated for 1 h at 37°C. After three washes with PBS-T, 100 µl of substrate solution (5 mg of o-phenylenediamine in 25 ml of 0.1 M citrate-phosphate buffer pH 5.0 plus 10 µl of 30% H_2_0_2_) was added to each well, and the reaction was interrupted after 8 min in the dark by the addition of 50 µl of 4 N H_2_SO_4_. The optical density was read at 492 nm with a Tecan Sunrise 96 well Microplate Reader (Tecan, Grödlg, Austria).

The same sera were tested by ELISA using the classical exoantigen from *P. brasiliensis* B339 (AgPbB339) at 10 µg/ml as determined by checkerboard titration.

### SDS-PAGE and western blotting

For the immunoblot study we used CFA from *P. lutzii* strain EPM 208 (10 µg/lane) and CFA from *P. brasiliensis* B339 (10 µg/lane). Sera that reacted with these two different antigens were compared. The gels and reagents for SDS-PAGE were prepared as described previously [31]; a 10% resolving gel and 3% acrylamide stacking gel were used. After electrophoresis, the gels were stained with Coomassie Blue solution (0.1% Coomassie Blue, 45% methanol, and 10% glacial acetic acid), and excess stain was removed with destaining solution (10% glacial acetic acid and 10% methanol). We selected 12 sera from patients with PCM due to *P. lutzii* previously confirmed by ID using CFA from *P. lutzii* strain EPM 208 and 12 sera from patients with PCM due to *P. brasiliensis* previously confirmed by ID using AgPbB339 and gp43 as antigens. For Western blot, the samples were transferred to nitrocellulose membranes (Milipore) according to Towbin et al. [26]. Gel contents were electrotransferred to nitrocellulose membranes at 400 mA for 1 h in a Trans-Blot cell (Bio-Rad) containing transfer buffer (25 mM Tris-HCl, 192 mM glycine, and 20% methanol [vol/vol]; pH 8.3). Free binding sites in the membranes were blocked by incubation for 2 h in 5% (wt/vol) non-fat dry milk in PBS-T (pH 7.5). Membranes were sliced vertically and the strips were incubated for 1 h at room temperature with diluted serum (1∶500 in PBS-T containing 5% non-fat milk; PBS-T-M). The strips were washed in PBS-T-M four times for 10 min each. The membranes were incubated with peroxidase-conjugated goat anti-human IgG (Sigma) at 1∶1,000 dilution for 1 h and then washed as above. The reactive antigenic molecules were developed by chemiluminescence mix reagents (Millipore, WBKLS0500) onto the membrane which is reactive to the conjugated secondary antibody peroxidase and luminol. To image the blot, we placed the transferred membrane in the transluminator UVITEC imager (Uvitec Cambridge, United Kingdom). The software settings in the Allience 4.7 software were set up to take several images from different time exposures, starting at 2 seconds with a total of ten images between 2 seconds. Then we selected the best time exposure from those 10 pictures taken.

### Statistical analysis

Performance measured analyzed for each test were: sensitivity, specificity, positive predictive value (PPV) and negative predictive value (PNV). The receiver operating characteristics (ROC) curve was drawn to determine the sensitivity and specificity for each antigen preparation (CFA, EXO and TCA) in ID test. The areas under ROC curves (AUC) were calculated to evaluate the diagnostic values of each antigen preparation. We assumed a test without diagnostic power when the ROC curve was linear with AUC of 0.5 (the ROC curve will coincide with the diagonal). A powerful test would give an AUC around 1.0, demonstrating the absence of both false positives and false negatives (the ROC curve will reach the upper left corner of the plot). To measure the degree of concordance of the results of the different assays, the kappa statistic and its 95% confidence interval (95% CI) were calculated. Kappa values were interpreted as follows: 0.00–0.20, poor agreement; 0.21–0.40, fair agreement; 0.41–0.60, moderate agreement; 0.61–0.80, good agreement; 0.81–1.00, very good agreement [32]. A p value of <0.05 was considered to indicate statistical significance. All statistical calculations were performed with the MedCalc Statistical Software version 13.2.0 (MedCalc Software bvba, Ostend, Belgium; http://www.medcalc.org; 2014).

## Results

**Figure 3:**
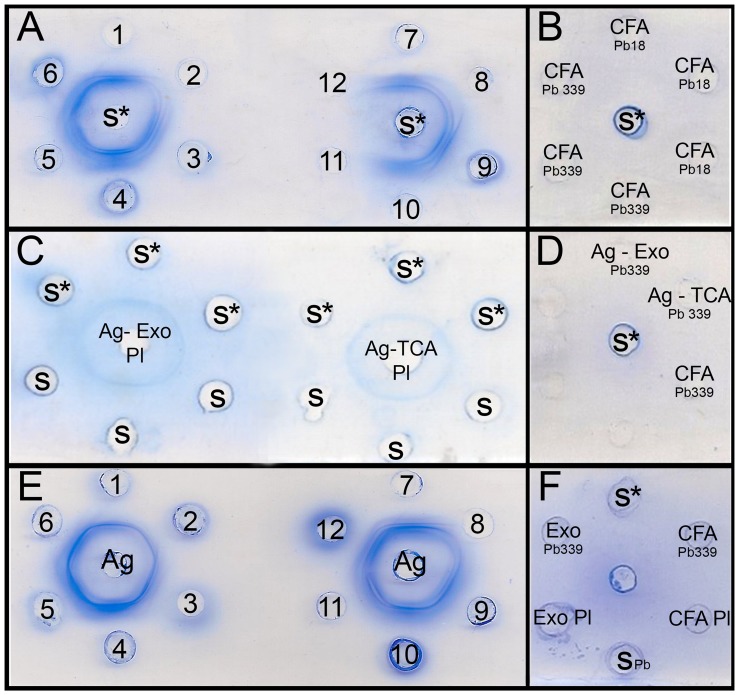
A) Immunodiffusion tests with different CFAs from 12 strains of *Paracoccidioides lutzii*. 1 = EPM 147; 2 = EPM 148; 3 = EPM 196; 4 = EPM 201; 5 = EPM 205; 6 = EPM 206; 7 = EPM 208; 8 = EPM 212; 9 = EPM 213; 10 = EPM 223; 11 = EPM 209; 12 = EPM 226. In the center well, S* = *P. lutzii* serum (gold standard). *P. lutzii* strains EPM 227 and EPM 228 were positive (not shown). **B**) Immunodiffusion test showing that S* = *P. lutzii* gold standard serum do not react with CFA from *P. brasiliensis* Pb18 and B339. **C**) Immunodiffusion test using Ag Exo (crude exoantigen of *P. lutzii*) and Ag TCA (crude exoantigen of *P. lutzii* precipitated with trichloroacetic acid) versus *P. lutzii* serum (S* = *P. lutzii* gold standard serum and S = suspected *P. lutzii* serum). Observe the weak band in both cases. **D**) immunodiffusion test showing that S* (*P. lutzii* gold standard serum) do not react with Ag Exo, Ag TCA and CFA from *P. brasiliensis* B339 antigen. **E**) Immunodiffusion test of 12 sera from patients with PCM due to *P. lutzii* using *P. lutzii* CFA. In the center well is the CFA antigen (Ag), and different PCM sera are in the peripheral wells (1 to 12). **F**) Immunodiffusion test showing that S* (*P. lutzii* gold standard serum) do not react with Exo or CFA of *P. brasiliensis* B339; and S_Pb_ (serum of PCM patient due to *P. brasiliensis*) do not react with Exo or CFA of *P. lutzii*.

Suspected *Paracoccidioides* spp. isolates were subjected to *HSP70* gene amplification. All clinical isolates had positive amplification and were identified as *P. lutzii* (Figure S1). During pilot studies, various *P. lutzii* CFA preparations were tested by ID; three were not reactive (Fig. 3A). *P. lutzii* strain EPM 208 was chosen for the CFA preparation based on the good and rapid growth of this strain, as well as the good bands observed on the slide. Fig. 3B shows that antigens (CFA) from *P. brasiliensis* Pb18 and B339 do not react with *P. lutzii* serum.

**Figure 4:**
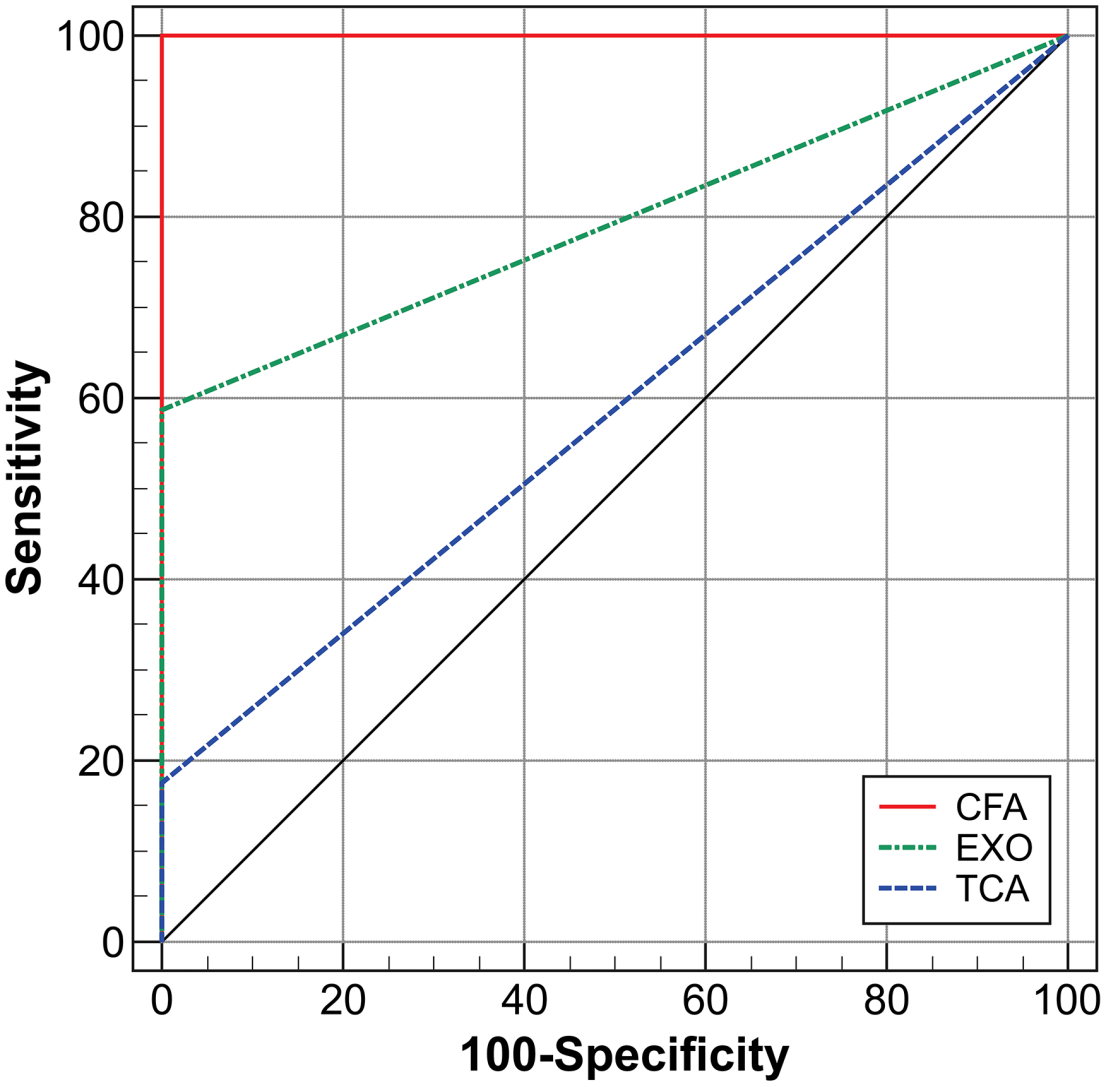
Receiver operating characteristics (ROC) curve for the CFA, EXO and TCA antigens derived from *Paracoccidioides lutzii* EPM208 isolate. The ROC is plotted between the true-positive rate (sensitivity) on the y-axis, and the false-positive rate (100-specificity) on the x-axis. The area under the curve (AUC) represents the accuracy of the ID test which was 1.0, IC 0.95–1.0, p<0.0001 for the CFA, 0.74±0.06, IC 0.68–0.87, p<0.0001 for the EXO and 0.58±0.04, IC 0.47–0.69, p = 0.0641 for the TCA antigens. The higher AUC above 0.5, the better the test.

In ID tests, 59 (66.2%) of 89 patient sera were reactive to the classical antigen AgPbB339, indicating that these sera were from patients with PCM due to *P. brasiliensis*. The 30 sera that were not reactive were re-tested with *P. brasiliensis*-gp43 purified antigen, and 13 (43.3%) were reactive, indicating that they were also from patients with PCM due to *P. brasiliensis*. Some of these serum samples were positive only for gp43 when tested with different protein concentrations of gp43 (Figure S2). The 17 non-reactive sera were re-tested with three antigenic preparations from *P. lutzii* strain EPM 208. Ten (58.8%) of the sera were reactive to the exoantigen preparation, but a weak precipitated band was observed (Fig. 3C). Only 3 (17.6%) of the sera were reactive to TCA-precipitated antigen, resulting in bands of low intensity (Fig. 3C). Figure 3D shows that antigens from *P. brasiliensis* B339 (Ag Exo, TCA or CFA) do not reacted with serum from PCM patient due to *P. lutzii*. In contrast, all 17 (100%) sera were reactive to the CFA, resulting in sharp bands (1, 2 or 3 bands) (Fig. 3E). Figure 3F shows that antigen from Exo B339 or CFA B339 do not reacted with serum from PCM patient due to *P. lutzii* and that antigen from Exo-PI or CFA-PI do not reacted with serum from PCM patient due to *P. brasiliensis*. Among those 17 reactive sera with CFA *P. lutzii*, 2 (11.7%) of them also reacted weakly with *P. brasiliensis* CFA antigen, indicating cross-reactivity between these two species. The most sensitive antigenic preparation was CFA-Pl, with a sensitivity 100% (IC 80.5–100) and specificity 100% (IC 94–100), followed by Exo-Pl with a sensitivity 58.82% (32.9–81.6) and specificity 100% (IC 94–100) and TCA-Pl with a sensitivity 17.65% (3.8–43.4) and specificity 100% (IC 94–100). Positive and negative predictive values (PPV, NPV) in ID assay were higher for CFA-Pl (PPV = 100, IC 79.4–100; NPV = 100, IC 94–100), followed by Exo-Pl (PPV = 100, IC 66.3–100, NPV = 89.55, IC 79.6–95.6) and TCA-Pl (PPV = 100, IC 15.8–100; NPV = 22.07, IC 70.3–89.2). Heterologous sera and serum samples from healthy individuals (negative control) did not react with the tested antigens. We used the area under the ROC curve (AUC) to evaluate the discriminatory values of the antigens (comparing subjects with PCM due to *P. lutzii* and those without the disease). Our results showed that the CFA-Pl antigen afforded better AUC values (AUC = 1.0, IC 0.95–1.0, *p*<0.0001) than those for the Exo-Pl (AUC = 0.74±0.06, IC 0.68–0.87, *p*<0.0001) and TCA-Pl (AUC = 0.58±0.04, IC 0.47–0.69, *p* = 0.0641) (Fig. 4). Judging from the deviating performance of different antigen preparations in ID assays we discarded the Exo-Pl and TCA-Pl antigens in subsequent experiments.

**Figure 5:**
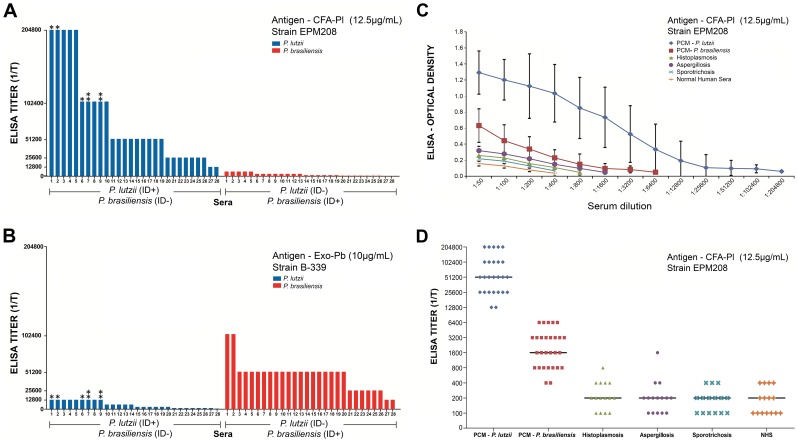
A) Individual serum ELISA titers (1/T) of 28 sera from patients with PCM due to *P. lutzii* (blue) compared to individual serum ELISA titers (1/T) of 28 sera from patients with PCM due to *P. brasiliensis* (red) reacting with Ag CFA-Pl EPM 208 (12.5 µg/ml). The *P. lutzii* sera presented ID + for *P. lutzii* CFA antigen and ID − for *P. brasiliensis* exoantigen (AgPbB339). The group of *P. brasiliensis* sera presented ID + for AgPbB339 antigen and ID − for *P. lutzii* CFA antigen. (*) represents *P. lutzii* gold standard serum and (**) represents sera that had a very weak cross reaction with *P. brasiliensis* exoantigen. **B**) Individual serum ELISA titers (1/T) of 28 sera from patients with PCM due to *P. lutzii* (blue) compared to individual serum ELISA titers (1/T) of 28 sera from patients with PCM due to *P. brasiliensis* (red) reacting with Ag Exo Pb B339 (10 µg/ml). The *P. lutzii* sera presented ID + for *P. lutzii* CFA antigen and ID − for *P. brasiliensis* exoantigen (AgPbB339). The group of *P. brasiliensis* sera presented ID + for AgPbB339 antigen and ID − for *P. lutzii* CFA antigen. (*) represents *P. lutzii* gold standard serum and (**) represents sera that had a very weak cross reaction with *P. brasiliensis* exoantigen. The same group of sera was used in A and B. **C**) ELISA median curves from sera from patients with PCM due to *P. lutzii*, PCM due to *P. brasiliensis*, and heterologous sera such as histoplasmosis, aspergillosis, sporotrichosis, and normal human serum. Antigen used was *P. lutzii* CFA (Ag CFA-Pl EPM 208 at 12.5 µg/ml). **D**) ELISA individual serum titers (1/T) of patients with PCM due to *P. lutzii*, *P. brasiliensis*, and heterologous sera such as Histoplasmosis, Aspergillosis, Sporotrichosis and normal human sera. By the end titer we could distinguish sera of PCM patients due to *P. lutzii* from sera of PCM due to *P. brasiliensis* and other mycotic diseases. Antigen used was *P. lutzii* CFA (Ag CFA-Pl EPM 208 at 12.5 µg/ml). Bars indicate the median.

For ELISA, the CFA from *P. lutzii* strain EPM 208 showed excellent reactivity for antibody detection using PCM sera (*P. lutzii* and *P. brasiliensis*) and heterologous sera (histoplasmosis, aspergillosis and sporotrichosis) and normal human sera as negative control. Figure 5A shows the individual serum titers of patients with PCM due to *P. lutzii* (sera # 1 to 28) and *P. brasiliensis* (sera # 29 to 58). Among *P. lutzii* sera, # 1, 2, and 6 represent the gold standard sera and sera from patients lacking treatment; 3, 4, 5, and 7 to 19 represent sera from patients who received 6 to 12 months of therapy; and 20 to 28 represent sera from patients with more than 12 months of therapy. Figure 5B shows these same groups of sera reacting with exoantigen from *P. brasiliensis* B339 (traditional antigen: AgPbB339). Figure 5C shows the median curve of 28 PCM sera (*P. lutzii*) compared to the median curve of 28 PCM sera (*P. brasiliensis*) and heterologous sera (histoplasmosis, aspergillosis and sporotrichosis), besides the NHS used as negative control. Figure 5D shows the results of the same set of sera but expressed by the titers of each serum. The heterologous sera and NHS always showed much lower titers in relation to the PCM sera. On an overall, we observed that the cross-reactivity with heterologous sera and NHS does not interfere with the interpretation of results with homologous sera and *P. lutzii* CFA antigen. Most of the sera from patients with PCM due to *P. lutzii* presented with titers from 1∶12,800 to 1∶204,800 (median = 51,200), whereas sera from patients with PCM due to *P. brasiliensis* presented with maximum titers of 1∶6.400 (median = 1∶ 1,600), indicating a great difference between the sera. Among the heterologous sera the maximum of cross-reactivity was 1∶1,600 for aspergillosis (median = 1∶200), 1∶800 for histoplasmosis (median = 1∶200), 1∶400 for sporotrichosis (median = 1∶200). NHS reacted until 1∶400 (median = 1∶200).

**Figure 6:**
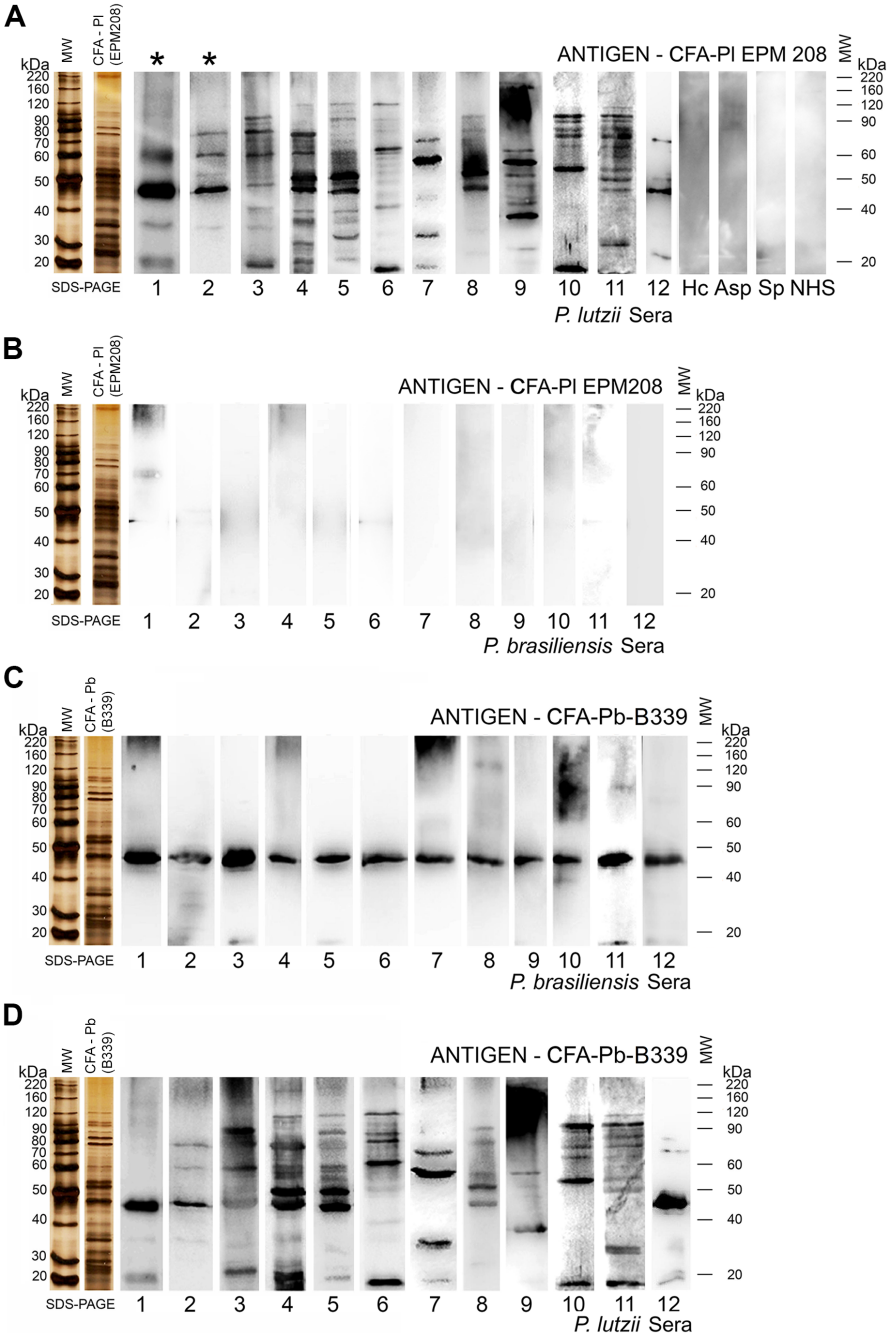
A) Western blot assays showing the reactivity of sera from patients with PCM due to *P. lutzii* versus *P. lutzii*-CFA antigen. Hc, Asp, Sp and NHS: representative blots of heterologous sera and normal human sera (15 sera each). (*) = represents *P. lutzii* gold standard serum. **B**) Reactivity of sera from patients with PCM due to *P. brasiliensis* versus *P. lutzii*-CFA antigen. We could distinguish *P. brasiliensis* from *P. lutzii* sera, as only sera from patients with PCM due to *P. lutzii* recognize antigenic molecules from *P. lutzii*-CFA. **C**) Western blot assays showing the reactivity of sera from patients with PCM due to *P. brasiliensis* versus *P. brasiliensis*-CFA (strain B339). Gp43 is basically recognized by PCM sera due to *P. brasiliensis*. **D**) Reactivity of sera due to *P. lutzii* versus *P. brasiliensis*-CFA (strain B339).

Western blot allowed us to distinguish PCM due to *P. lutzii* and due to *P. brasiliensis*, as only sera from patients with PCM due to *P. lutzii* are able to recognize antigenic molecules from *P. lutzii*-CFA (Fig. 6A). Sera from patients with PCM due to *P. brasiliensis* did not recognize any *P. lutzii* CFA antigens (Fig. 6B). In addition, we used Kappa test to assess the agreement between different serological assays (ID, ELISA and Western blot) based on CFA-Pl antigen. Kappa test (*k* = 1) revealed a perfect agreement between all pairwise comparisons for different assays.

In order to verify cross-reactions between both *Paracoccidioides* species, sera from patients with PCM due to *P. brasiliensis* and due to *P. lutzii* were tested against CFA from B339 (Fig. 6C and 6D).The results showed that sera from PCM due to *P. brasiliensis* recognized only gp43 molecule; sera from PCM patients due to *P. lutzii* recognized various molecules. Considering the main bands evidenced, the reactivity were almost similar to that obtained with *P. lutzii* sera and CFA-Pl.

## Discussion

The World Health Organization (WHO) estimates that more than 1 billion people, one-sixth of the world's population, suffer from one or more neglected diseases. The diseases are most heavily concentrated in low-income nations in Africa and Latin America, and the most common types of neglected diseases are tropical diseases. Many neglected tropical diseases are caused by parasites, which are spread by insects or contact with contaminated water or soil (http://www.who.int/neglected_diseases/diseases/en/). In this scenario, PCM can be considered a neglected disease. Coutinho et al. [2] analyzed 3,181 deaths from PCM in Brazil based on 16 years of sequential data (from 1980 to 1995). During this period, PCM showed considerable magnitude and low visibility, representing the eighth most common cause of death from predominantly chronic or recurrent types of infectious and parasitic diseases. The study showed that the mortality rate justifies classifying this disease as an important health problem in Brazil. However, no government programs exist for this mycosis, with rare punctual exceptions.

For a century PCM was thought to be caused solely by *P. brasiliensis*, but recent studies [11], [12], [18]–[21], [33] have shown that other species of *Paracoccidioides* can also cause PCM. These findings reveal that problems in the serological diagnosis of PCM were due to an incorrect use of antigens from the genus, as only antigens of *P. brasiliensis* (PS3) have been used for this purpose. With the discovery of *P. lutzii*, it became clear that new antigens from these new species would be needed to obtain more accurate diagnoses. Recently, a fatal case of PCM due to *P. lutzii* fungemia was reported [34] and other two cases were reported in the North region of Brazil [23].

Until recently, the serological diagnosis of PCM by ID could be accomplished using only the standard exoantigen from B339, which has a high concentration of gp43 antigen, the immunodominant and specific molecule in ID tests. Early suspicions that this serology was not entirely certain were raised by our group [35] when we observed that an exoantigen produced from an isolate (Pb550B) from the Central-West region of Brazil was capable of reacting with a larger number of PCM sera from that region than the standard antigen from an isolate from São Paulo in the Southeast region (AgPbB339). Nevertheless, when tested with PCM sera from São Paulo, the antigen from the Central-West region of Brazil did not produce satisfactory results. Also, when we used the standard antigen AgPbB339, it reacted very well with sera from São Paulo, but very poorly with sera from the Central-West region. Analyses of antigens obtained from the B339 and 550B isolates showed that the former produced high levels of gp43, which was undetected in the 550B filtrate. This variation in gp43 expression likely influences the low reactivity observed in ID tests using sera from patients from the Central-West region of Brazil. At that time, nothing was known about *P. lutzii*; therefore, we suggested using regional strains to aid in the diagnosis of PCM for the Central-West region. Importantly, B339 belongs to the S3 species, whereas isolate 550B is from the Central-West region of Brazil, where *P. lutzii* is more frequently found.

The main fact that spurred this study was the observation that sera from patients with PCM (previously proven by direct examination of secretions or histopathological analysis) were negative by conventional serology using the standard AgPbB339 antigen in ID tests. In order to elucidate the problem with serological diagnosis, we developed a strategy. First, all sera from patients suspected of PCM should be tested against the traditional exoantigen of *P. brasiliensis* (AgPbB339) and purified gp43 molecule derived from the same standard strain. The reactive sera were then considered to be PCM due to *P. brasiliensis*. The non-reactive sera were then tested against CFA from *P. lutzii* EPM 208.

Applying this strategy, we were able to diagnosis 72 patients with PCM due to *P. brasiliensis*. The remaining non-reactive patients (n = 17) were diagnosed mostly by ID using CFA from *P. lutzii* and also by ELISA and Western blot. This type of antigen (CFA) is very easy to prepare and is very useful in the diagnosis of PCM due to *P. lutzii*, exhibiting clearly visible precipitation bands. Among these 17 sera, 2 of them also reacted with the traditional exoantigen B339, but with a weak reactivity, indicating cross reaction between these two species of *Paracoccidioides*. However, the exoantigen preparation (from *P. lutzii*) also managed to obtain positive reactions, but the precipitation line was very weak, making it difficult to read the slides. In addition, the antigenic preparations obtained by TCA precipitation were useful, but the bands were quite weak. In both later antigens, the sensitivity of the reactions was lower than that obtained with the CFA preparation.

Thus, the CFA preparation obtained from *P. lutzii* EPM 208 was the better antigen for use in ID tests to diagnosis PCM due to *P. lutzii* from the Central-West region of Brazil. Other *P. lutzii* strains were tested with similar results; however, among 14 CFA preparations, three were unable to precipitate antibodies in sera from *P. lutzii*-PCM patients, indicating that antigenic differences exist among these strains. Therefore, more studies are necessary to elucidate the antigenic variability among *P. lutzii* strains. Another curiosity we observed when we tested CFA preparations from *P. lutzii* in the ID tests was that better results were obtained when the protein concentrations of the antigen were greater than 1200 µg/ml. Protein concentrations less than 1200 µg/ml resulted in very weak bands, hindering the visualization of precipitation reactions. Machado et al. [36] described CFA as being inefficient for the diagnosis of PCM due to *P. lutzii*.

In ELISA, sera from patients with PCM due to *P. lutzii* presented with higher antibody titers than those obtained for the sera from patients with PCM due to *P. brasiliensis* which can perfectly differentiate between patient sera from *P. brasiliensis* and *P. lutzii*. Heterologous sera, such as histoplasmosis, aspergillosis and sporotrichosis and normal human sera exhibited very low cross-reactivity and did not interfere with interpretations of the main system. It was expected that the sera from PCM patients due to *P. brasiliensis* reacted more intensively with *P. lutzii* antigens because these two species belong to the same genus. Certainly, the antigenic components present in *P. lutzii* are constituted by specific antigens of this species plus common antigens shared with *P. brasiliensis*. Probably, antibodies elicited during infection by *P. brasiliensis* are only generated against antigens from this species whereas antibodies elicited during infection by *P. lutzii* are generated by specific antibodies against antigens in this species and also against antigens shared with *P. brasiliensis*. It was also expected that sera from patients with PCM due to *P. brasiliensis* reacted more intensively than heterologous sera (histoplasmosis, aspergillosis and sporotrichosis), and not almost in the same level. Perhaps, these results may be explained by the presence of common antigens among *P. brasiliensis* and heterologous fungi, which are reacting in this ELISA system.

In relation to the strategy used for immunoblotting in the present study, we found that sera from patients with PCM due to *P. brasiliensis* do not recognize any antigen from *P. lutzii* CFA. In contrast, sera from patients with PCM due to *P. lutzii* were able to recognize antigens from both antigenic preparations (CFA from *P. lutzii* or *P. brasiliensis*). Therefore, *P. lutzii* is antigenically more complex. These findings suggest that *P. lutzii* has its own species-specific antigens and antigens common with *P. brasiliensis*. Thus, we can distinguish between sera from patients with PCM due to *P. brasiliensis* and those with PCM due to *P. lutzii*. However, the specific *P. lutzii* antigen still needs to be identified. The reactivity of PCM sera due to *P. brasiliensis* predominantly to gp43 of its homologous antigen may be explained by the fact of the great immunogenicity of this molecule, so that, the immune response to other minor molecules is not evidenced.

Based on our results, we estimate that the incidence of PCM due to *P. lutzii* is much greater than we imagine, even though the Central-West region of Brazil where the disease is prevalent has not invested in acquiring new tools for a specific diagnosis of this entity. In general, laboratories for the diagnosis of infectious diseases are small, have few resources, and perform a limited number of tests per day. Our strategy meets all of the requirements for use in laboratories for the diagnosis of PCM due to *P. lutzii*, as well as seroepidemiological studies. In addition, the test does not require special skills and can be used for a small number of samples. Although the cost of ELISA and Western blot is higher than that of ID, they are economical if the costs associated with laboratory personnel, quality control, and reagent storage are taken into account.

Surely *P. lutzii* is not confined to the Central-West region of the country; we have described two cases in the northern region (Pará State) [23], and we have strains of *P. lutzii* isolated in the Rondônia State (North region) and Paraná (South region) in our fungal collection. With our proposed strategy for the diagnosis of PCM caused by *P. lutzii*, many new cases may arise throughout Brazil and other South American countries. Studies are underway in our laboratories to identify the species-specific antigen for *P. lutzii* in order to use it for simple and specific diagnosis of this mycosis.

## Supporting Information

Figure S1 **PCR for molecular identification of** ***P. lutzii*** **strains.** The primers amplify *HSP70* only in *P. lutzii*. M = molecular weight standard in bp. 1 = EPM 147; 2 = EPM 148; 3 = EPM 193; 4 = EPM 201; 5 = EPM 205; 6 = EPM 206; 7 = EPM 208; 8 = EPM 209; 9 = EPM 212; 10 = EPM 213; 11 = EPM 226; 12 = EPM 223; 13 = EPM 227; 14 = EPM 228; 15 = B339 (*P. brasiliensis*). C - = negative control reaction. Note: EPM 147 is Pb01 (*P. lutzii*, classical strain) and the positive control.
(TIF)

Figure S2 **Immunodiffusion tests showing the reactivity of three sera from patients with PCM due to** ***P. brasiliensis*** **with different concentrations of gp43.** A) Serum S1 reacted with gp43 at 500 ng. B) Serum S2 reacted with gp43 at 250 ng. C) Serum S3 reacted with gp43 at 31 ng. Most of the sera reacted very well at 4, 2, or 1 µg of gp43 per well (data not shown).
(TIF)

Scheme S1 **Immunodiffusion slide scheme.** Left, disposition of the different antigenic preparations for the diagnosis of PCM due to *P. brasiliensis*. Right, disposition of the antigens for diagnosis of PCM due to *P. lutzii*. AgPbB339 = classical antigen for diagnosis of PCM by *P. brasiliensis*; gp43 = purified antigen from exoantigen AgPbB339, specific fraction for PCM by *P. brasiliensis*; CFA-PI (EPM 208) = cell free antigen from *P. lutzii* strain EPM 208.
(TIF)
