# Supplementary figures and images for: Serology of Paracoccidioidomycosis Due to Paracoccidioides lutzii
Source: PLoS Negl Trop Dis. 2014 Jul 17;8(7):e2986. doi: 10.1371/journal.pntd.0002986 (PMC4102441; doi:10.1371/journal.pntd.0002986)

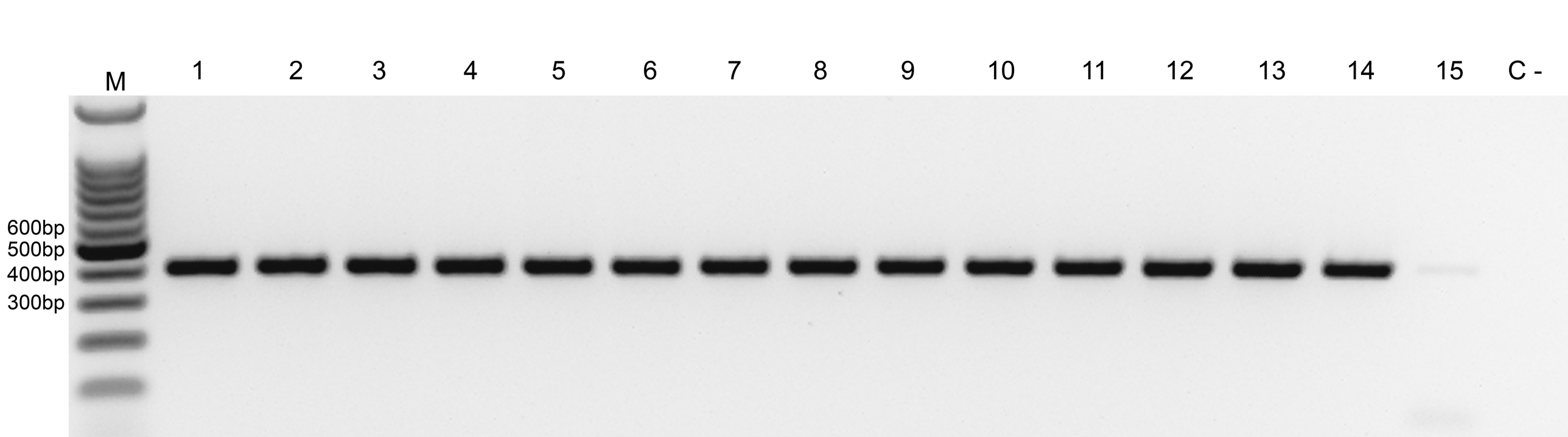

Supplement: Figure S1 — PCR for molecular identification of P. lutzii strains. The primers amplify HSP70 only in P. lutzii. M = molecular weight standard in bp. 1 = EPM 147; 2 = EPM 148; 3 = EPM 193; 4 = EPM 201; 5 = EPM 205; 6 = EPM 206; 7 = EPM 208; 8 = EPM 209; 9 = EPM 212; 10 = EPM 213; 11 = EPM 226; 12 = EPM 223; 13 = EPM 227; 14 = EPM 228; 15 = B339 (P. brasiliensis). C - = negative control reaction. Note: EPM 147 is Pb01 (P. lutzii, classical strain) and the positive control. (TIF) [file pntd.0002986.s001.tif]

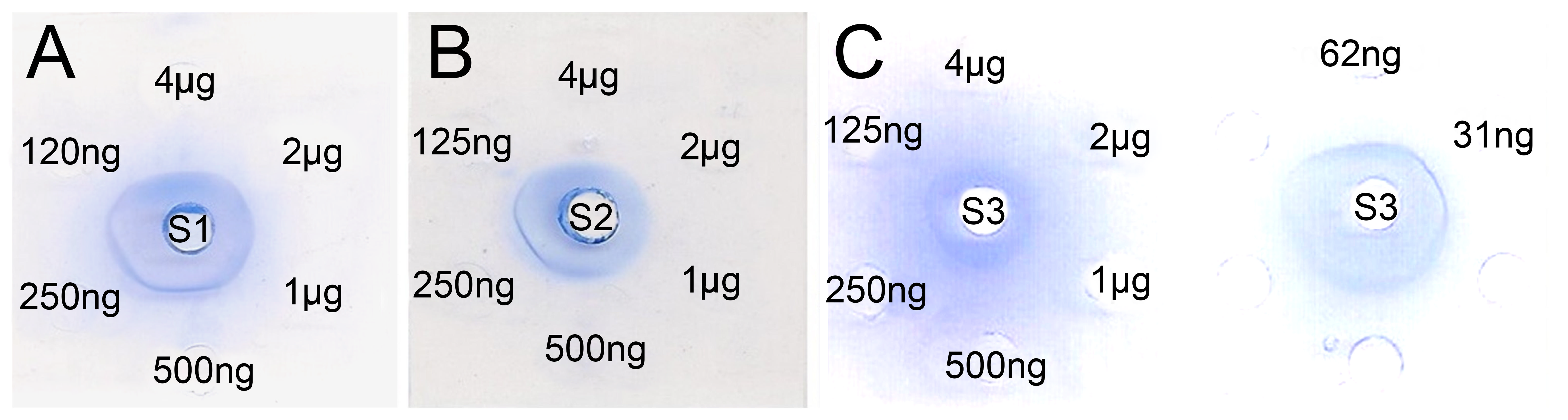

Supplement: Figure S2 — Immunodiffusion tests showing the reactivity of three sera from patients with PCM due to P. brasiliensis with different concentrations of gp43. A) Serum S1 reacted with gp43 at 500 ng. B) Serum S2 reacted with gp43 at 250 ng. C) Serum S3 reacted with gp43 at 31 ng. Most of the sera reacted very well at 4, 2, or 1 µg of gp43 per well (data not shown). (TIF) [file pntd.0002986.s002.tif]

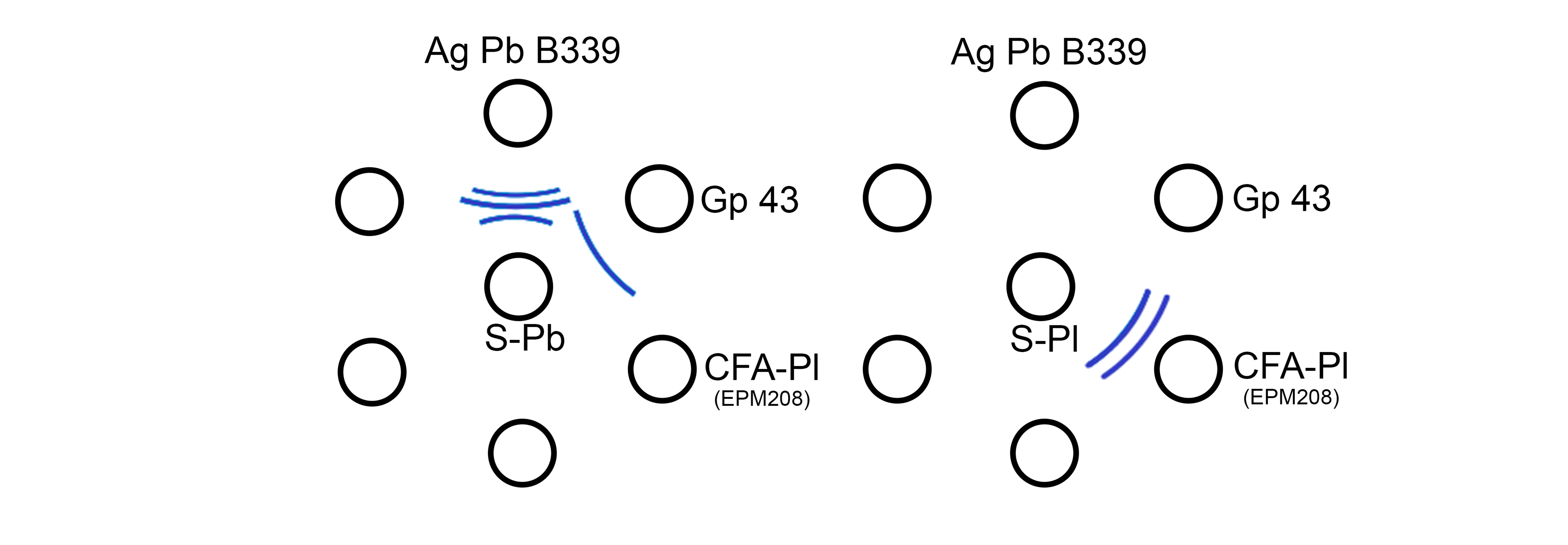

Supplement: Scheme S1 — Immunodiffusion slide scheme. Left, disposition of the different antigenic preparations for the diagnosis of PCM due to P. brasiliensis. Right, disposition of the antigens for diagnosis of PCM due to P. lutzii. AgPbB339 = classical antigen for diagnosis of PCM by P. brasiliensis; gp43 = purified antigen from exoantigen AgPbB339, specific fraction for PCM by P. brasiliensis; CFA-PI (EPM 208) = cell free antigen from P. lutzii strain EPM 208. (TIF) [file pntd.0002986.s003.tif]
